# Perspectives of the Obstetric Care Environment for Pregnant Individuals Who Have an Opioid Use Disorder

**DOI:** 10.1089/whr.2024.0142

**Published:** 2025-02-05

**Authors:** Samantha Girasulo, Caro Maltz, Maggie Weichert, Joy S. Kaufman, Amanda Mele, Karen Hunkele, Kimberly A. Yonkers, Nancy Byatt, Ariadna Forray

**Affiliations:** ^1^ Department of Psychiatry, Yale School of Medicine, New Haven, Connecticut, USA.; ^2^ Renaissance School of Medicine, Stony Brook University, Stony Brook, New York, USA.; ^3^ Department of Psychiatry, University of Massachusetts Chan Medical School, Worcester, Massachusetts, USA.

**Keywords:** opioid use disorder, patient perspectives, perinatal obstetric care, qualitative study

## Abstract

**Objective:**

To assess the specific barriers and facilitators for pregnant individuals who have an opioid use disorder (OUD) receiving perinatal care.

**Methods:**

We conducted key informant interviews with patients who received care from obstetric clinicians who had been trained to provide medication for opioid use disorder (*n* = 16). We asked patients about the care they received for their OUD, the quality of communication with their perinatal care team, and any recommendations for improving OUD care. Two staff independently coded transcripts, and we used content analysis to identify themes.

**Results:**

Our analysis resulted in three main facilitators that support participants receiving care from their obstetric clinician: (1) positive relationship with supportive and nonjudgmental clinician; (2) access to medication for opioid use disorder (MOUD); and (3) access to therapeutic and peer supports. Patients noted that nonjudgmental clinicians provided a care environment where they felt safe, did not experience stigma, and felt they could be active participants in their care. Patients also expressed that access to MOUD and clinical and supportive services were beneficial components of perinatal care. The main barriers identified included lack of access to transportation, long wait times for treatment programs, and difficulty accessing MOUD.

**Conclusions:**

The results of this study suggest that increased obstetric provider education about OUDs and providing trauma-informed care for pregnant individuals who have an OUD may help reduce barriers to accessing care and increase satisfaction with care for this population. Furthermore, the present study suggests obstetricians provide in-house access to MOUD, if possible, or assist patients with referrals to care, as these may reduce the structural barriers patients face.

## Introduction

Between 2010 and 2017, the rate of pregnant individuals who have maternal opioid-related diagnoses increased from 3.5 to 8.2 per 1000 deliveries.^1^ While medication for opioid use disorder reduces overdose fatalities and enhances quality of life,^2^ many pregnant individuals with opioid use disorder (OUD) have difficulties accessing medication treatment for OUD (MOUD).^3^ One such challenge is that providers of outpatient MOUD are less likely to treat pregnant compared to non-pregnant individuals (75% vs. 91%).^4^^,5^ Additionally, pregnant individuals with OUD experience delayed and reduced rates of prenatal care in contrast to those with other substance use disorders.^6^ Financial barriers, inadequate social support, and stigma deter pregnant people with OUD from substance use disorder treatment.^7^ Stigma and discrimination are also associated with increased risk of not adhering to prescribed MOUD, discontinuation of MOUD,^8^ and return to use.^9^ Individuals with OUD subjective experience of perinatal care include feeling disrespected by the professionals serving them, receiving inconsistent care, and having limited access to health and social services.^10^ Conversely, supportive and non-judgmental care, peer and family support, and proper medication management contribute to adherence to OUD treatment.^11,12^

Recent research provides insight into the experiences of pregnant people with OUD as it pertains to adherence and retention in opioid-related care,^9,13^ the labor and birth experience and general experiences during the perinatal period.^11,12,14^ However, patients’ experiences of receiving prenatal care in conjunction with OUD treatment are not well understood. Given that there is little research aimed at gathering patient insights for improving the prenatal care experience for individuals with OUD, this study sought to elucidate the perspective of perinatal individuals with OUD receiving MOUD in obstetric settings and their recommendations regarding ways to improve this care.

## Methods

Data were collected at baseline from pregnant individuals enrolled in Project SMART (Support Models for Addiction Related Treatment), a matched-pair cluster randomized clinical trial with 12 obstetric practices comparing two healthcare models to increase the support of pregnant and postpartum individuals with OUD.^15^ As part of the SMART study, at least one clinician from participating obstetric practices became certified to prescribe MOUD. Pregnant patients enrolled in the larger study (*n* = 16) with a history of OUD and receiving care from practices in the study were invited to share their perspectives of the obstetric care provided and recommendations to improve this care. The interview participants were randomly chosen from our study sites. Data collection occurred from January 2020 to January 2023. A team of five research personnel from our study including an investigator, three research assistants, and a project manager conducted 60-minute qualitative interviews *via* an encrypted Zoom call. Demographic characteristics for the study participants can be found in Table 1.

**Table 1.** Demographics

	Percent	Count
Mean age (range)	33.56 [26–40]	
Gender		
Female	100%	16
Race		
Caucasian, White	75%	12
Mixed biracial	13%	2
Other	6%	1
Unknown, prefer not to say	6%	1
Ethnicity		
Spanish origin, Hispanic, or Latina	13%	2
Not of Spanish origin, Hispanic, or Latina	88%	14
Marital Status		
Living with partner	56%	9
Never married	25%	4
Married	13%	2
Prefer not to answer	6%	1
Education		
Some high school	19%	3
High school diploma or GED	25%	4
Associate degree	6%	1
Some college	38%	6
Bachelor’s degree or beyond	19%	3

The research team developed the interview protocol based on their clinical experiences and previous research. The protocol was also vetted by the Patient Stakeholder Group for Project SMART. Participants were asked about their perceptions of OUD, the care they received for their OUD, whether having an OUD influenced the quality of their care, the quality of communication with their perinatal care team, and any recommendations for improvement of OUD care. All participants consented to participate in the main study and subsequently provided verbal consent for this interview. All interview recordings were transcribed and de-identified for analysis. Participants received a $50 gift card for their time. The Institutional Review Board at Yale University approved this study; Clinicaltrials.gov number: NCT04240392.

### Data analysis

The study team used content analysis^16^ to examine the transcripts and identify emerging themes. All interviews were transcribed verbatim from the audio recordings into Microsoft Word documents. The research team coded one transcript together, clarified the operational definitions of the codes, and made modifications to the codes. Revisions were made to the codebook as new themes emerged. Two researchers coded each transcript and met to reach consensus. A senior researcher reviewed the coded transcripts to confirm agreement with the codes. After coding was completed, the team identified themes endorsed by three or more participants and selected representative quotes to exemplify the themes. The use of a team of researchers to code transcripts and the iterative process of data collection and analysis, was employed to increase the credibility, transferability, dependability, and confirmability of the findings.^17,18^

## Results

Analysis of the transcripts resulted in three main themes related to aspects of the OUD care participants received from their obstetric providers: (1) positive relationship with supportive and nonjudgmental obstetrical provider; (2) access to medication for opioid use disorder (MOUD); and (3) access to therapeutic and peer supports.

### Positive relationship with supportive and nonjudgmental obstetrical provider

Patients reported feeling that when their obstetrical provider communicated in an informative, empathetic way, it facilitated feelings of trust and safety. (*n* = 6)

“I don’t have any problems with asking her for help with anything. She’s, like I said, she’s easy to talk to. She’s kind, compassionate. She doesn’t judge me. So, like if I have any kind of issue, I know I can openly speak to her about it and that, like I said, she’ll help me and steer me in the right direction.”

Study participants reported that feeling safe with their obstetrical providers resulted in an increase in two-way communication. As well, patients indicated that receiving educational information from their provider led to improved health.

“There’s a lot of knowledge that I didn’t have prior that I did need that I didn’t know I needed. There’s tests that I didn’t know existed. There’s a lot out there that they- I’ve just recently found out and learned.”

When asked how providers could improve care for pregnant patients with an OUD, participants recommended that providers treat patients with OUD as they would treat any patient coming into their practice. They noted that this could be accomplished by being supportive and nonjudgmental (*n* = 7).

“So, you know we obviously have some issues that occurred in our life that brought us to where we’re at, but you know just treat us the same as everyone else and you know that would be a lot more helpful for someone with the substance abuse disorder than if we’re treated differently.”

Participants also recommended that increased education for providers about OUD would provide a more positive experience for patients (*n* = 10).

“The best thing that they could give us is to have to go through a mandated educational class on addiction, and really understand addiction, to really be able to understand the people who are pregnant and are currently in the whirlwind of addiction. And how to be a little sensitive to those people.”

“I think there’s always going to be stigma about addictions. There’s not really much that we could do to help with that besides, like I said, just get them educated.”

The majority (*n* = 13) of participants reported that when they were involved in their health care decisions they felt empowered and more comfortable communicating with their providers.

“Like, sometimes people think, oh, cause, you’re an addict, you’re like an idiot, or you don’t know what’s going on or, you know, they look down upon you. My OB is not like that. Like, she gives me 100% control pretty much of what’s gonna be going on or wants my opinion, 100%, you know, which makes me feel good too.”

### Access to medication for opioid use disorder

Medication for opioid use disorder was reported as a beneficial component of obstetric treatment and for patients to maintain sobriety (*n* = 9).

“It’s definitely helped me to continue to stay clean. It’s been kind of a support in my life to balance me out so to say where you know maybe I’m able to deal with life and what life throws at me a lot better, a lot more stable, so those are definitely I would say the key ways that it’s really helped me.”

“Well, I definitely believe that it [MOUD] can help reduce cravings. If you’re sick and hurting you’re not gonna be ready for any kind of counseling or anything like that, and what it does, is it really kind of stabilizes you. If somebody has been doing opioids for, you know, 20 years it’s not gonna be easy. So, it’s kind of a stepping down stone. I think for pregnant women especially, so they do not use any other street drugs, it’s very helpful.”

Participants reported that receiving MOUD from their obstetric provider was beneficial, as obstetrical providers are entrusted with keeping the pregnant person and fetus safe (*n* = 6).

“I like that my [obstetric] provider is actually the person that’s prescribing my medication. I actually like it that way ‘cause then it’s involved in my pregnancy and she sorta knows everything that’s going on so I don’t have to update her every time I see her. She knows exactly what’s going on so I think it should be the main role basically.”

Some patients (*n* = 4) indicated that receiving MOUD from their obstetric provider eliminated the need to go elsewhere for medication and reduced the burden on them.

“Well, it’s one less doctor you have to see one less clinic you have to go to someone and so forth, you know. That can definitely get complicated, but I think they could also probably closely monitor the patient and, you know, the care that they’re getting a little bit more than then someone going to a different clinic.”

“…it felt disconnected because I was getting my meds from one place and my OB care from another, and it can be helpful—especially for people that don’t have transportation or a whole lot of time—to get everything done in one place.”

Others felt that obstetric providers prescribing MOUD increased the burden on the provider (*n* = 4).

“So I think they should continue to help and watch and observe and make sure that you’re OK. And do urine tests and stress tests and all that. But when it comes to it - you just don’t want to put too much on top, like they go through so much and deal with a lot.

All participants who reported that obstetric providers should not prescribe MOUD in practice, as it would increase provider burden, believed that these providers are still obligated to connect patients with MOUD by providing referrals (*n* = 4).

“They shouldn’t become a clinic, but I feel like they should help you. If there’s someone not in a clinic already, like getting help for suboxone or methadone, they should let them know the right treatments for pregnancy or help them get into clinic.”

### Therapeutic and peer supports

Patients reported that having access to mental health treatment (*n* = 9) and/or peer support (*n* = 3) was beneficial, especially if they were able to access these treatments through their obstetrical practice or the practice facilitated referral to these services and supports (*n* = 7).

“You need all the support, AA, NA, just to have people around you that are sober and doing the right thing that you can relate to, talk to, all of that, makes a huge difference in an addict’s life, 100%.”

“I like that they [obstetric provider] have groups where I go so I can talk to other women that are sort of in the same position or similar position that I’m in.”

Some participants reported that a supportive provider, access to medication, and access to support were most effective in combination (*n* = 4).

“It’s a combination, yeah, for sure. One resource is not gonna really do, you have to work with many and I don’t have a lot of close family. I mean I do have my family, I am very blessed that I have my mom, my son, and my father is supportive. But you know, that’s really it. And there are people that don’t even have that. You really need to have a team behind your back.”

Engaging in mental health and medication treatment facilitated patients’ feelings of safety for themselves and their fetus, however, many informants reported experiencing barriers to accessing the support they needed (*n* = 7). Some barriers reported include lack of access to transportation, long wait times for treatment programs, and difficulty accessing MOUD.

“I had to wait a full week [to talk to the **MOUD** clinic]. And then when I did talk to them, they’re like ‘Oh we don’t treat pregnant women.’ So that was definitely an issue.”

“Transportation, yeah, and finding time, especially if they work.”

## Discussion

The perceptions of the participants in this study are consistent with previous research which demonstrated that access to treatment based on the needs of pregnant individuals with an OUD is lacking.^19,20^ Participants in the present study report that some barriers to care, including access to MOUD, mental health treatment, and peer support, could be reduced, ideally, through providing these services within obstetric practices. However, if provisioning these services in the office is not possible, providers should facilitate and support referrals to community-based services. Though both of these options for assisting patients with OUD in accessing care are understood by the patients of this study to increase provider burden, the latter option was also agreed to be the least patients would expect of their providers.

The pregnant individuals from this study report that they prefer to receive care from obstetric clinicians who offer unbiased communication and promote feelings of safety during prenatal appointments. In order to facilitate this type of care, obstetric clinicians would benefit from implementing trauma-informed care in which each patient is approached without judgment, recognizing they each have unique needs.^21,22^ The American College of Obstetricians and Gynecologists (ACOG) advocates for providers to integrate trauma-informed care into their practice with the goal of improving maternal health outcomes.^21^ The ACOG indicates that obstetric providers are “uniquely positioned to be leaders in building clinical environments that are emotionally and physically safe for patients and staff,”^21^ and advocate for creating space for dialogue and avoiding stigmatizing patients, mirroring the recommendations made by these study participants.

This study also revealed that pregnant individuals with an OUD want to be involved in decisions about their prenatal care. This could be accomplished through shared decision-making by providing patients with details about treatment options and their benefits and risks. The use of a shared decision-making tool has been shown to help patients feel empowered about their treatment choices.^23^

Increased education on the provision of prenatal care for patients with an OUD may also help to reduce the barriers to treatment identified in this study. Providers could access continuing education such as the Alliance for Innovation on Maternal Health (AIM) Care for Pregnant and Postpartum People with Substance Use Disorder.^24^ which focuses on substance use and obstetrics, enabling clinicians to stay current on recommended models of care and increase the quality of care provided.^24^ Results from this study are also consistent with recommendations made in the AIM Care for Pregnant and Postpartum People with Substance Use Disorder Patient Safety Bundle.^25^ Similar to the recommendations made by the individuals in this study, the AIM Safety Bundle advocates for providers to develop and maintain referral resources and communication pathways between obstetric providers and community-based organizations providing services and supports for pregnant individuals who have an OUD.^25^ Other education options for providers that are effective in increasing referrals to treatment and facilitating patient-provider communication include practice-wide implementation of models such as Project ECHO.^26^ or Collaborative Care.^27^ These models place emphasis on providing support for patients and continuing education for providers.^15^

The present study has some limitations. The sample size and the collection of data from two Northeast states may limit the generalizability of the findings. However, the results and recommendations made by study participants are consistent with what has previously been found in the literature and in practice guidelines. Based on our results, implications for future research would suggest the need to develop and test more education and technical assistance opportunities (*e.g.,* consultation on the implementation of practice guidelines) for obstetric clinicians.

The purpose of this study was to understand the perspectives of perinatal individuals with OUD who are receiving MOUD in obstetric settings and their recommendations for improving this care. Participants reported that facilitating access to MOUD and to therapeutic and peer support alongside a supportive and non-judgmental relationship with patients increased trust in their provider and the care they receive. While the recommendations about the provision of care that were made by study participants may increase the burden on providers, they have the potential to reduce barriers to care for pregnant individuals with OUD and increase the quality of the prenatal care experience for these patients.

## Abbreviations Used

AA: Alcoholics Anonymous
MOUD: medication for opioid use disorder
NA: Narcotics Anonymous
OB: obstetrician
OUD: opioid use disorder
